# Sexual hormones monitoring in surface waters and wastewaters from Northern Italy by thin film microextraction coupled with HPLC–MS/MS

**DOI:** 10.1007/s11356-024-34306-6

**Published:** 2024-07-17

**Authors:** Francesca Merlo, Valentina Quarta, Andrea Speltini, Antonella Profumo, Clàudia Fontàs, Enriqueta Anticó

**Affiliations:** 1 Department of Chemistry, University of Pavia, Via Taramelli 12, 27100 Pavia, Italy; 2 Department of Chemistry, University of Girona, C/ Maria Aurèlia Capmany 69, 17003 Girona, Spain

**Keywords:** Endocrine disrupting compounds, Oestrogens, Environmental water samples, Thin film microextraction, Polymeric membranes, Sample treatment

## Abstract

The occurrence of sex steroid hormones, viz. oestrogens and progestins, in aquatic ecosystems is of global concern due to their role as endocrine disrupting chemicals, even at low concentration (μg L^−1^ or less). Thus, it is essential to monitor these organic pollutants to get a realistic picture of their presence and to control their contamination levels in environmental water bodies. In this respect, we have explored the use of self-prepared polymeric films as novel sorptive phase for the microextraction of 17β-estradiol, 17α-ethinylestradiol, estrone, progesterone, medroxyprogesterone acetate and hydroxyprogesterone. The thin film microextraction procedure has been developed, evaluating different film compositions, sample volumes and elution conditions to recover the sorbed analytes. The overall method provides good reproducibility (RSD < 12%) and recoveries higher than 60%. The final method has been applied to environmental monitoring in surface waters (river and lake samples) and urban wastewater treatment plant effluents and influents from Northern Italy, to get a contamination snapshot of this highly urbanized area.

**Supplementary Information:**

The online version contains supplementary material available at 10.1007/s11356-024-34306-6.

## Introduction

In recent years, oestrogens and progestins have been categorized as endocrine disrupting compounds (EDCs) as they can interfere with the endocrine system of exposed organisms in the environment, negatively affecting their development and physiology, even at low concentration (μg L^−1^ or less). Exposure to ECDs may impair neurological and immune systems as well as cause reproductive and developmental effects in humans and animals (Kabir et al. 2015). It has been suggested that the increase in cases of attention deficit/hyperactivity disorder, premature puberty, obesity and endocrine cancers may be linked to ECDs (Schug et al. 2011). Intentional intake of anabolic–androgenic steroids or doping among athletes is prohibited partially because of the associated side effects such as gynecomastia, hypospadias, smaller testis, infertility and cancer (Basaria 2010). Thus, the occurrence of sex hormones in aquatic ecosystems is of global concern also due to their continuous input from human activities (Bilal et al. 2021; Goeury et al. 2022; Ismanto et al. 2022). Steroidal hormones are excreted from animals and humans in partially metabolized or relatively unmetabolized form. As a result, they are inevitably incorporated into the water bodies via inadequately treated wastewater, disposal of animal wastes in freshwater or runoff into groundwaters (Bilal et al. 2021; Zhang et al. 2021; Ciślak et al. 2023). Indeed, oestrogens and progestins are widely used for both animal and human treatments, for example in the treatment of menstrual-disorders, such as hormone replacement therapy, and in oral contraceptives. 17α-Ethinylestradiol (EE2) is the most common oestrogen in birth control pills, often in combination with progesterone (PROG), while natural hormones as estrone (E1) and 17β-estradiol (E2) are released in great quantities by mammals (Bilal et al. 2021; Goeury et al. 2022; Ciślak et al. 2023). The consumption of these compounds is expected to increase in the coming years as a result of improvements in health standards, especially in developing countries (Olasupo and Suah 2021).

Furthermore, when EDCs are present in the form of a mixture — as in the case of oestrogens and progestins due to their co-formulation — a ecotoxicity higher than that of the single compound has been observed due to the combined effects (Riva et al. 2019). Because of the serious environmental risks associated with their diffusion and widespread use, European environmental quality standards (EQS) have been established in water bodies for E2 (0.4 ng L^−1^) and for E1 (3.6 ng L^−1^), which have been included together with 17α-ethinylestradiol (EE2) in the 1st and the 2nd Watch Lists of compounds to be monitored across the EU in the field of water policy (European Commision (EC) 2015, 2018).

Despite their well-known toxicity, fate and occurrence, monitoring studies in surface waters and wastewaters are limited (Meffe and de Bustamante 2014; Riva et al. 2019; Castiglioni et al. 2020). This is a consequence of the high cost of analysis, and the time and labour involved in the analytical methods for the sensitive determination of these contaminants (Olasupo and Suah 2021; Sacdal et al. 2020). This highlights the need for easy and sensitive methods to get a realistic picture of the presence of these emerging pollutants. Both liquid- and solid-phase extraction techniques have been proposed to achieve this goal, but the application of miniaturized procedures that meet the requirements of Green Sample Preparation (GSP) (López-Lorente et al. 2022) has not been so deeply investigated (Merlo et al. 2023). In this context, thin-film microextraction (TF-ME) is an efficient sample preparation technique consisting in the introduction of a thin film sheet in the sample with enhanced extraction rate and broad application scopes by the design of different sorptive phases (Seidi et al. 2019). Polymer inclusion membranes (PIMs), composed of a polymer and a plasticizer to provide flexibility, have been explored in sample preparation for the preconcentration of inorganic species and organic pollutants (Almeida et al. 2017; Olasupo and Suah 2021). Some key features of PIMs are their versatility, ease and low cost of preparation, resulting in an advantageous material for TF-ME. In our previous works (Vera et al. 2018; Merlo et al. 2022), we have demonstrated that membranes made of a bio-polymer derivative (cellulose triacetate, CTA) and a plasticizer can be successfully applied to the TF-ME of organic pollutants, *viz*. pesticides and personal care products. However, to the best of our knowledge, the use of PIMs for analytical purposes in TF-ME has not been described yet. The aim of this work is to develop a microextraction procedure followed by liquid chromatography coupled to tandem mass spectrometry (LC–MS/MS) for the concurrent determination of three oestrogens (E2, EE2, E1) and three progestins (PROG, M-PROG, H-PROG). Thus, the polymeric membrane made of CTA as polymer and dibutyl sebacate (DBS) as plasticizer has been used to extract these compounds in surface waters (river and lake samples) and urban wastewater treatment plant (UWWTP) effluents and influents from Northern Italy. This study provides a snapshot of the pollution scenario in a highly populated area in Europe.

## Experimental section

### Chemicals and materials

All chemicals were reagent grade or higher in quality. Solid standards of standards of E2, E1, EE2, PROG and M-PROG were supplied by Sigma-Aldrich (Milan, Italy) and analytical grade H-PROG standard was purchased from Steroids (Cologno Monzese, Italy). Molecular structures, molecular weight and Log*P* values are shown in Fig. S1.1 (Appendix 1, Supplementary Material).

Sex hormones stock solutions (1000 mg L^−1^) were prepared in methanol (MeOH) and stored in the dark (4 °C). Intermediate solutions (1.25 mg L^−1^, 0.250 mg L^−1^) were prepared in methanol by dilution from a multiple solution (25 mg L^−1^), and they were renewed weekly.

HPLC gradient grade MeOH, ACN, ultrapure water, ammonium fluoride (NH_4_F, for ACS analysis) and analytical grade salts (NaHCO_3_, Na_2_SO_4_, CaCl_2_ •2 H_2_O) were acquired from Carlo Erba Reagents (Milan, Italy).

The polymer CTA (Fluka, Buchs, Switzerland) was used for the membrane preparation. Chloroform (CHCl_3_) from Panreac (Castellar del Vallès, Spain) was used to dissolve CTA. The plasticizers used were nitrophenyl octyl ether (NPOE) from Sigma-Aldrich (Sant Louis, Missouri, USA), and DBS from Fluka (Buchs, Switzerland). Their structure and physico-chemical properties are shown in Fig. S1.2 (Appendix 1, Supplementary Material).

### Thin film microextraction coupled with the HPLC–MS/MS method

#### Thin film microextraction procedure

According to the solvent casting method, two different membranes were herein prepared, both containing 70% (w/w) of CTA and 30% (w/w) of plasticizer, NPOE or DBS (see molecular structures and physico-chemical properties reported in Fig. S1.2, Appendix 1, Supplementary Material), resulting in the films labelled as F1 (DBS) and F2 (NPOE). The preparation and characterization of the polymeric membranes were deeply described in previous studies (Vera et al. 2018; Merlo et al. 2022). Preliminary studies were undertaken in tap water to evaluate the sorption capability of F1 and F2 towards oestrogens and progestins. Tap water has been selected due to its similarity to environmental waters, in terms of pH, ionic strength, and its invariant composition over time. A piece of each membrane was immersed in water sample (40 mL) enriched with 50 μg L^−1^ of each analyte under orbital agitation (Stuart orbital shaker SSL1, Cole-Parmer) at 250 rpm for different extraction times, ranging from 30 min up to 24 h. Aliquots (200 μL) were withdrawn at fixed time and the amount of analyte remained in aqueous solution was determined by HPLC–MS/MS (multiple reaction monitoring -MRM- mode) to evaluate the kinetic and to calculate the extraction efficiency (EE%) according to the following equation:

1 $$EE\%=\left(1-\frac{{A}_{t}}{{A}_{t0}}\right)\times 100$$

where A_t0_ is the peak area at *t* = 0 and *A*_*t*_ is the peak area after a predetermined contact time.

Subsequent trials (40 mL, 5 μg L^−1^ of each analyte under orbital agitation for 4 h) were performed to study the elution step. MeOH and ACN, both compatible with CTA polymer, were tested for ultrasound-assisted elution, evaluating also the influence of time (15 and 5 min) and solvent volume (2 and 1 mL). The eluate was analysed in HPLC–MS/MS (MRM mode) to calculate the efficiency of the elution step in terms of recovery (R%) using the following Eq. (2):

2 $$R\%= \frac{amount \;of \;eluted \;compound \;(ng)}{initial \;amount \;of \;compound \;in \;water \;(ng)}\times 100$$

The standard addition on the TF-ME eluate was selected to obtain the amount of eluted compound.

Recovery was evaluated for 40-, 70- and 100-mL sample volume, in tap water from the municipal waterworks, simulated river water (prepared by dissolving 0.168 g NaHCO_3_, 0.0355 g Na_2_SO_4_, 0.1642 g CaCl_2_ •2 H_2_O in 1 L pure water), and the effluent of the UWWTP, in contact with F1.

#### Chromatographic separation coupled with mass spectrometry detection

The instrumental analysis was performed with an Agilent (Cernusco sul Naviglio, Italy) HPLC apparatus 1260 Infinity coupled with an Agilent 6460C MS spectrometer ESI–MS/MS system. The chromatographic column was an Agilent Zorbax Eclipse Plus C18 (2.1 mm × 150 mm, 3.5 μm) equipped with an Ascentis C18 guard column (2.1 mm × 2 mm, 5 μm), both thermostated at 30 °C (± 0.8 °C).

The mobile phase was (A) NH_4_F 1 mM in water and (B) ACN. The gradient profile was adapted to the analytes by shortening one already present in the literature (Bianchini et al. 2023) and started with 30% B; then, the percentage of B was increased to 85% in 3 min and held for 8 min; and further increased to 98% in 0.5 min and held for 1 min as washing step. The initial conditions (30% B) were returned by 7-min equilibration time. The flow rate was 0.5 mL min^−1^, and the sample injection volume was 10 μL.

The ESI source parameters were drying gas (N_2_) temperature 350 °C; drying gas flow 12 L min^−1^; nebulizer 50 psi; sheath gas temperature 400 °C; sheath gas flow 12 L min^−1^; capillary voltage 4000 V positive and 3000 V negative; nozzle voltage 0 V positive and 1500 V negative; cell accelerated voltage (CAV) 4 V positive and 1 V negative (Bianchini et al. 2023). The acquisition was performed in MRM mode, using two transitions for each compound as reported in Table S1.1 (Appendix 1, Supplementary Material). A typical MRM chromatogram of a standard solution (25 μg L^−1^ of each analyte in MeOH) is shown in Fig. S1.3 (Appendix 1, Supplementary Material).

The diverter valve was set to prevent loss in sensitivity excluding the highly polar matrix components that are eluted with the solvent peak and the highly retained matrix components that are washed off the column with higher percentage of organic mobile phase, sending to the ionization only the eluate from the column in the time range 7.5–11.1 min. To avoid any possible carry-over and loss of sensitivity, pure MeOH was injected between analyses.

### Analytical evaluation of TF-ME coupled with HPLC–MS/MS

The TF-ME procedure coupled with HPLC–MS/MS quantification was intra-laboratory validated for lake, river and wastewater samples according to the main analytical figures of merit.

The in-sample matrix-matched calibration method was applied, spiking the water samples in the concentration range 0.010–0.500 μg L^−1^ for each analyte. Method detection and quantification limits (MDLs and MQLs, respectively) were calculated as three and ten times the ratio between the baseline noise away from the peak tail and the regression line slope (Sheehan and Yost 2015).

The matrix effect (ME%) was calculated as following:

3 $$ME\%= \frac{response \;in \;eluate}{response \;in \;pure \;solvent}\times 100$$

Accuracy was appraised by recovery tests in water samples (e.g. Mergozzo Lake, simulated river samples and UWWTP effluent, see Table S2.1, Appendix 2, Supplementary Material) enriched at different concentration levels (25, 40, 100 ng L^−1^) and maintained in contact with F1.

### Monitoring campaign: analysis of water samples from Northern Italy

In the final TF-ME procedure coupled with HPLC–MS/MS, the square film piece was put in contact with 70 mL of water sample (native pH) under orbital agitation at 250 rpm for 4 h. The elution was performed in ultrasound bath (Ultrasonic cleaner, 60 Hz, VWR, Italy) for 5 min with 1 mL of MeOH, and the eluate was analysed in HPLC–MS/MS (MRM mode) without any derivatisation. Different types of water samples were considered, namely surface waters from lakes in high impact tourist areas (Como and Garda in Lombardy) and rivers in urbanized areas (Tanaro in Piedmont, Ticino in Lombardy), and wastewater samples collected downstream a UWWTPs discharge point (Vigevano, Lombardy), and at the outlet of Pavia UWWTP (before and after UV treatment).

The area of study is reported in Appendix 2 together with physical–chemical parameters of the water samples collected (see composition in Tables S2.1-S2.2, Appendix 2, Supplementary Material).

## Results and discussion

### Thin film microextraction coupled with the HPLC–MS/MS method

In previous works, membranes made of 70% CTA as the polymer and 30% of plasticizers (NPOE or DBS) have been successfully employed for the extraction of organic pollutants (pesticides and personal care products, respectively) from environmental waters (Vera et al. 2018; Merlo et al. 2022). Thus, preliminary studies were undertaken in tap water enriched with 50 μg L^−1^ of each analyte (native pH, see Analytical evaluation of TF-ME coupled with HPLC–MS/MS and Table S2.1, Appendix 2, Supplementary Material) to evaluate the sorption kinetic of these films towards oestrogens and progestins, different classes of emerging organic contaminants. As expected, the amount of analytes extracted increased with time, achieving a plateau after 4 h for both membranes, hence it has been fixed as the extraction time. Both films exhibited satisfactory extraction capability (EE% > 60%), while the extraction efficiency was negligible (< 8%) for all analytes when a 100% CTA film was used (data not shown), further confirming the significant contribution of the plasticizer in the sorption process (Vera et al. 2018; Merlo et al. 2022). The lipophilic character of plasticizers (see Fig. S1.2, Appendix 1, Supplementary Material) guarantees the solubility of sex hormones in the membrane, and in the case of progestins, it is enhanced by π-stacking with the nitrophenyl group in NPOE (F2).

With the aim of developing an effective extraction method for the detection and quantification of the target analytes, the preconcentration by both membranes was studied in tap water (40 mL) enriched at lower concentration (5 μg L^−1^ of each analyte). Ultrasound-assisted elution (15 min) was preliminary selected to recover the analytes, according to previous works (Merlo et al. 2022; Quintanilla et al. 2023) and MeOH (2 mL), and was chosen as eluent. Table 1 shows the mean recovery values for each analyte, obtained by the standard addition method on the eluate to compensate for possible matrix effects.

**Table 1:** Mean recovery values (R%, *n* = 3) with standard deviation (Std. Dev.) achieved with DBS-based membrane (F1) and NPOE-based membrane (F2) in tap water (40 mL, 5 μg L^−1^ for each analyte)

	Mean recovery (%)	
	DBS-based membrane (F1)	NPOE-based membrane (F2)
E2	68 (9)	54 (5)
EE2	93 (14)	34 (1)
E1	81 (12)	51 (3)
PROG	84 (6)	88 (3)
M-PROG	87 (15)	89 (23)
H-PROG	63 (3)	38 (1)

It could be noted that NPOE-based membrane did not provide quantitative recovery due to an incomplete desorption in solvent suggesting that the analytes entrapped in this membrane are strongly retained. On contrast, the DBS-based membrane guarantees quantitative recoveries for all compounds; thus, it was selected as the most suitable film.

With the aim of reducing the analysis time, solvent waste and possible matrix effects, in line with the principles of GSP (criteria 4, 6, 8) (López-Lorente et al. 2022), the elution time was reduced from 15 to 5 min and the elution volume from 2 to 1 mL. No significant differences were observed, hence preserving the elution efficiency and enhancing the sensitivity. Next, different volumes (40, 70 and 100 mL enriched with 0.4 μg L^−1^ for each analyte) of water samples were also tested. The TF-ME was tested not only in tap water, but also in lake (Mergozzo Lake) and simulated river samples and UWWTP effluent (see Table S2.1, Appendix 2, Supplementary Material) with the aim of applying the method for environmental monitoring. An aliquot of each unspiked sample was simultaneously analysed, showing no peaks at the retention time of analytes, thus excluding the presence of both interferences and target compounds.

**Fig. 1:**
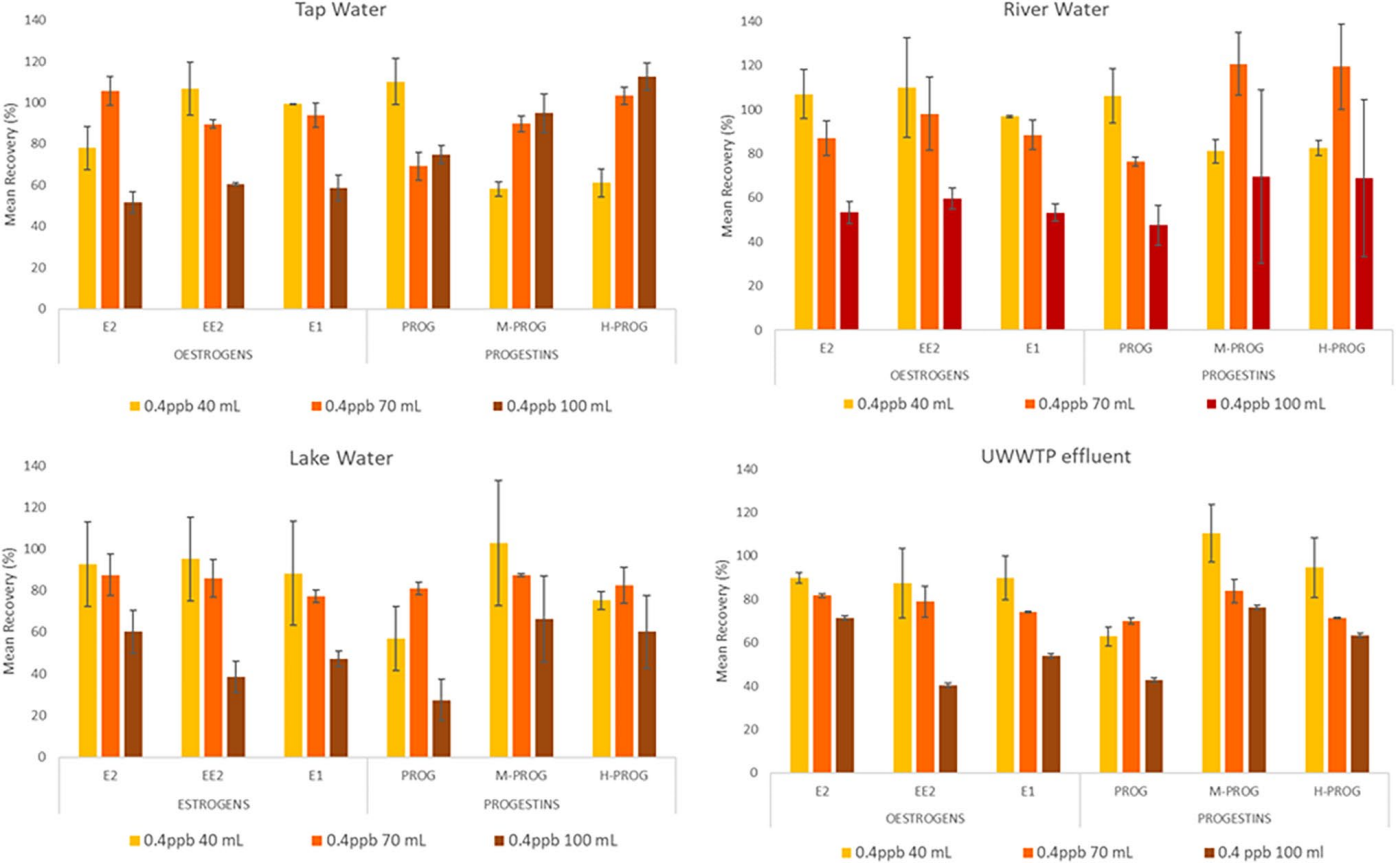
Mean recovery values (% R, *n* = 3) with standard deviation (Std. Dev.) achieved in tap, river, lake waters and UWWTP effluent (0.4 μg L^−1^ for each analyte) at different volumes (40, 70 and 100 mL)

It can be observed that the recovery values for each analyte remain stable among the studied matrices, as shown in Fig. 1. This is a remarkable achievement and highlights that the microextraction procedure is not affected by the composition of the matrix itself. In fact, when the complexity of the samples increases, the presence of organic and inorganic constituents could negatively affect the extraction of the target compounds, resulting in lower recoveries.

On contrast, a dependence on sample volume was noticed, with a drop in oestrogens’ recovery when processing 100-mL sample. This behaviour may be attributable to a low extractant surface/sample volume ratio; thus, this sample volume was excluded. On the basis of these results, 70 mL was chosen as the sample volume, as it provides a good compromise between recovery and enrichment factor (and therefore sensitivity).

### Analytical evaluation of TF-ME coupled with HPLC–MS/MS

The TF-ME method was intra-laboratory evaluated for lake, river, wastewater samples according to the main analytical figures of merit, as described in the “Analytical evaluation of TF-ME coupled with HPLC–MS/MS” section.

The matrix effect (ME%, see Eq. 3) was less than ± 20% for oestrogens in all aqueous matrices, on the contrary, a strong matrix effect was observed for progestins (see Tables S3.1, S3.2, S3.3, Appendix 3, Supplementary Material). This different behaviour could be ascribable to the different ionization mode of these classes, negative for oestrogens and positive for progestins. For river and wastewater samples, the signal of all the analytes was enhanced, and for lake samples both suppression (progestins) and enhancement (oestrogens) of signal was observed. Therefore, the in-sample matrix-matched calibration method was applied, spiking the water samples in the concentration range 0.010–0.500 μg L^−1^ for each analyte. A good correlation was found between the spiked concentration and peak area determined after elution, as shown in Tables S3.1, S3.2, S3.36 (Appendix 3, Supplementary Material). MDLs and MQLs were suitable for environmental monitoring of steroid sex hormones at the low nanograms per litre levels, in line with EQS, as pointed out in Table 2.

**Table 2:** MDL and MQL values (ng L^−1^) in water samples calculated from each matrix-matched calibration curve

	Lake water		River water		UWWTP effluent	
	MDL	MQL	MDL	MQL	MDL	MQL
E2	0.1	0.4	0.2	0.4	1.6	4.9
EE2	0.2	0.7	0.2	0.5	0.1	0.3
E1	0.2	0.4	0.1	0.3	0.1	0.4
PROG	2.2	6.8	1.6	4.7	3.0	9.0
M-PROG	0.4	1.3	0.1	0.3	0.6	1.8
H-PROG	5.7	17.4	1.8	5.4	3.5	10.6

Precision (expressed as relative standard deviation, RSD%) was lower than 12%, while trueness (expressed as recovery, R%) spanned from 61 to 116%, as pointed out in Table 3.

**Table 3:** Mean recovery values (R%, *n* = 3) observed in river, lake and UWWTP effluent samples, spiked at three different concentrations

		Mean recovery (%)							
	River water			Lake water			UWWTP effluent		
	100 ng L^−1^	40 ng L^−1^	25 ng L^−1^	100 ng L^−1^	40 ng L^−1^	25 ng L^−1^	100 ng L^−1^	40 ng L^−1^	25 ng L^−1^
E2	70	84	75	95	83	74	70	60	65
EE2	77	99	88	114	82	82	85	89	83
E1	79	108	81	89	94	90	75	102	83
PROG	101	92	90	102	90	95	83	87	85
M-PROG	70	61	78	71	63	105	94	62	70
H-PROG	71	116	77	82	71	90	74	99	82

All these values underline the applicability of the proposed method for monitoring these ECDs in surfaces waters and wastewaters.

### Monitoring campaign: analysis of water samples from Northern Italy

The final method was applied to the analysis of surface water and wastewater samples (see Monitoring campaign: analysis of water samples from Northern Italy for further details), all collected in the North of Italy in spring–summer 2023 (see Table 4).

**Table 4:** Sex hormones concentration (ng L^−1^) in waters samples from Northern Italy, quantified by the developed thin film microextraction

	Como Lake	Garda Lake	Ticino River (city centre)	Ticino River (upstream)	Ticino River (downstream)	Tanaro River	UWWTP effluent pre-UV	UWWTP effluent post-UV
E2	7.3	< MQL	< MDL	< MDL	< MDL	3.6	< MDL	< MDL
EE2	14.2	< MQL	< MDL	< MDL	< MDL	11.6	< MDL	< MDL
E1	8.7	0.6	< MDL	< MDL	1.5	2.5	1.2	< MDL
PROG	19	< MQL	< MQL	40	48	< MQL	91	73
M-PROG	12	1.5	< MQL	1.2	3.7	70	7.2	4.2
H-PROG	< MQL	< MQL	< MDL	< MDL	< MDL	< MDL	< MQL	< MDL

The results of this monitoring study across the water bodies from Northern Italy give an overall snapshot of the low but widespread actual contamination levels of these endocrine disruptors. The target contaminants were generally measured at concentrations from a few ng L^−1^ to tens ng L^−1^, in agreement with other monitoring studies from Northern Italy (Castiglioni et al. 2018, 2020; Riva et al. 2019; Merlo et al. 2020; Bianchini et al. 2023), with Ticino River (city centre) as the cleanest point in our study.

An anthropogenic contamination could be observed in the results of PROG and M-PROG, which were detected and/or quantified in more than 50% of water samples in this work at the highest values. To this sense, it is noteworthy the concentration levels found in the post-UV UWWTP effluent compared to the pre-UV, highlighting only a partial removal by the technology involved in water treatment and a greater contamination in respect to the data from 2018 (Merlo et al. 2020). They were also found in surface bodies, suggesting a release/entry from various sources, both urban and rural ones.

Interestingly, certain surface samples exceeded the threshold level for E2 (0.4 ng L^−1^) and E1 (3.6 ng L^−1^). Moreover, E1 is the most recurrently detected, also because it is not only produced during the menstrual cycles but also it is one of the degradation products of E2 (Bilal et al. 2021). Concentrations of oestrogens in Como Lake water were slightly higher than the ones found in 2017 and 2018 (Castiglioni et al. 2020), whereas levels in Garda Lake water were much lower than those found by our research group (Bianchini et al. 2023).

These findings highlight the importance of examining the whole mixture of these EDCs and contribute to improve the knowledge of their occurrence in a highly urbanized area of Europe, in the field of EU-wide monitoring programs. To this sense, the thin film microextraction is a very promising approach for environmental monitoring also for in situ applications, which can be useful to tackle spatial and temporal markers from anthropogenic impact.

## Conclusions

The use of polymeric membranes has proven to be an alternative and effective sorptive phase for the preconcentration of a type of endocrine disruptors in a wide field of real water samples. The developed thin film microextraction procedure was reliable in enriching sex hormones simultaneously with good time efficiency, low solvent consumption and good reproducibility. The results of a monitoring study of water bodies from Northern Italy give an overall picture of contamination, showing that concentrations of the measured compounds are generally low.

The thin film approach is very promising for environmental monitoring and may find a host of applications in the immediate future to facilitate the detection of these compounds and to support water policymakers.

## Supplementary Information

Below is the link to the electronic supplementary material.

Supplementary file1 (DOCX 142 KB)

## Data Availability

Data will be made available on request.
