# Supplementary material for: Sexual hormones monitoring in surface waters and wastewaters from Northern Italy by thin film microextraction coupled with HPLC–MS/MS
Source: Environ Sci Pollut Res Int. 2024 Jul 17;33(22):11140–7. doi: 10.1007/s11356-024-34306-6 (PMC13415270; doi:10.1007/s11356-024-34306-6)
Supplement: Supplementary file 1 — Supplementary file1 (DOCX 142 KB) [file 11356_2024_34306_MOESM1_ESM.docx]

***Supplementary Material***

**Sexual hormones monitoring in** **surfaces waters and wastewaters from Northen Italy by thin film microextraction coupled with HPLC-MS/MS**

Francesca Merlo^1^, Valentina Quarta^1^, Andrea Speltini^1^, Antonella Profumo^1^, Claudia Fontàs^2^, Enriqueta Anticó^2^*

*^1^Department of Chemistry, University of Pavia, via Taramelli 12, 27100 Pavia, Italy*

*^2^Department of Chemistry, University of Girona, 17003 Girona, Spain*

**Corresponding author:* enriqueta.antico@udg.edu

**Appendix 1: Experimental Part**

**Fig. S1.1.** Molecular structures and physico-chemical properties of the target analytes.


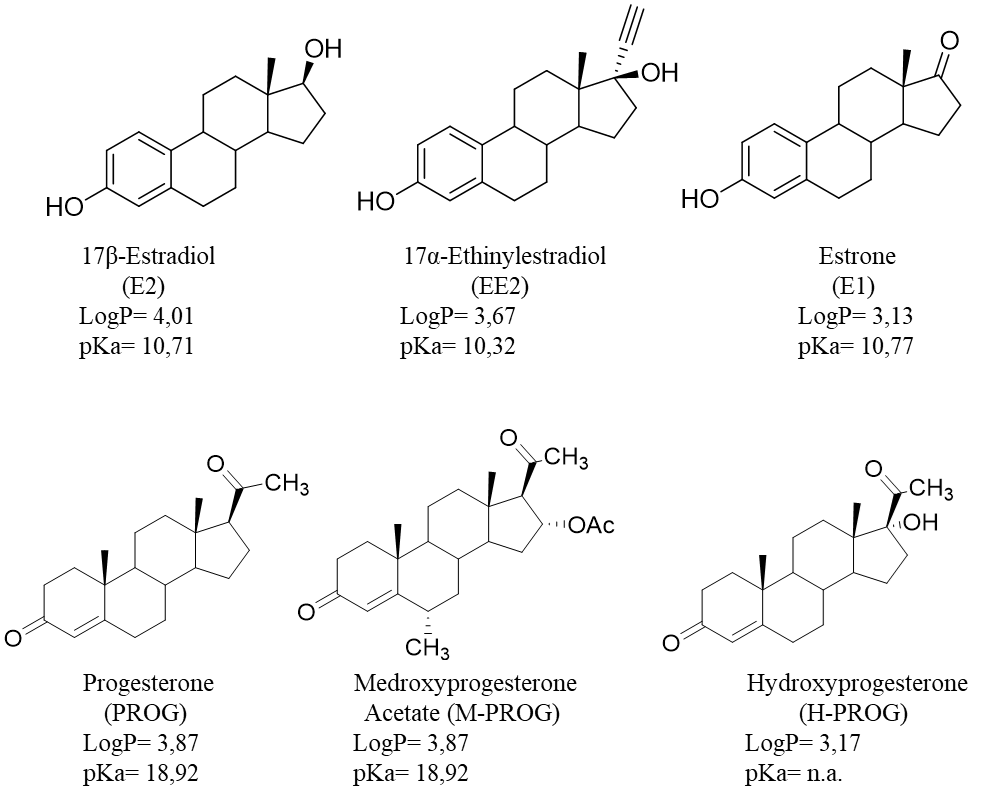


**Fig. S1.2.** Molecular structures and physico-chemical properties* of the target plasticizers.

**
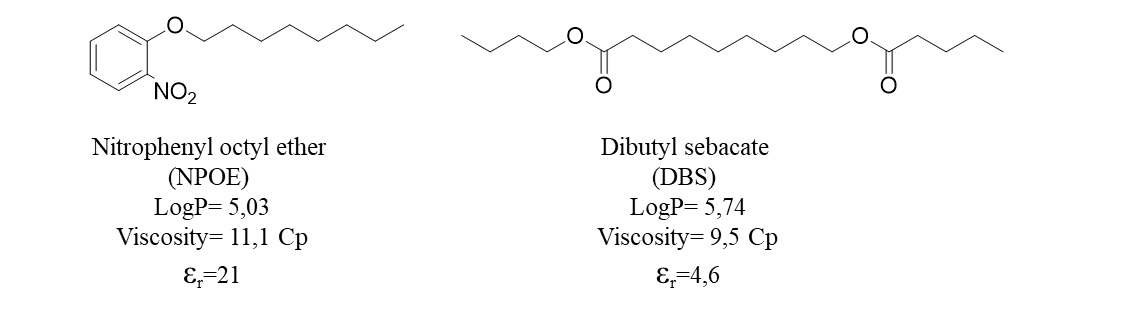
**

*Ref. Merlo et al., Microchemical Journal 175 (2022) 107120

**Table S1.1.** MRM conditions for the HPLC-ESI-MS/MS analysis.

| **Analyte** | **Precursor Ion (*m/z*)*** | **Product ion (*m/z*)** | **Dwell time (ms)** | **Fragmentor Energy (V)** | **Collision Energy (V)** | **Retention Time (min)** |  |
| --- | --- | --- | --- | --- | --- | --- | --- |
| E2 | 271 | 183.3 | 100 | 166 | 50 | 8.2 |  |
|  |  | 143.1 | 100 | 166 | 64 |  |  |
| EE2 | 295 | 145.1 | 100 | 154 | 44 | 8.4 |  |
|  |  | 143.1 | 100 | 154 | 68 |  |  |
| E1 | | 269 | 145.1 | 100 | 148 | 44 | 8.7 |
|  |  |  | 143.1 | 100 | 148 | 60 |  |
| PROG | 315 | 109 | 50 | 94 | 24 | 10.6 |  |
|  |  | 97 | 50 | 94 | 20 |  |  |
| M-PROG | 387 | 327.4 | 50 | 106 | 8 | 10.4 |  |
|  |  | 123.1 | 50 | 106 | 24 |  |  |
| H-PROG | 331 | 109.1 | 50 | 118 | 28 | 9.0 |  |
|  |  | 97.1 | 50 | 118 | 24 |  |  |

*[M-H]^-^ adduct for negative ions (oestrogens) and [M+H]^+^ adduct for positive ions (progestins).

**Figure S1.3.** Typical MRM chromatograms of a standard solution (25 μg L^-1^ of each analyte): a) oestrogens and b) progestins.


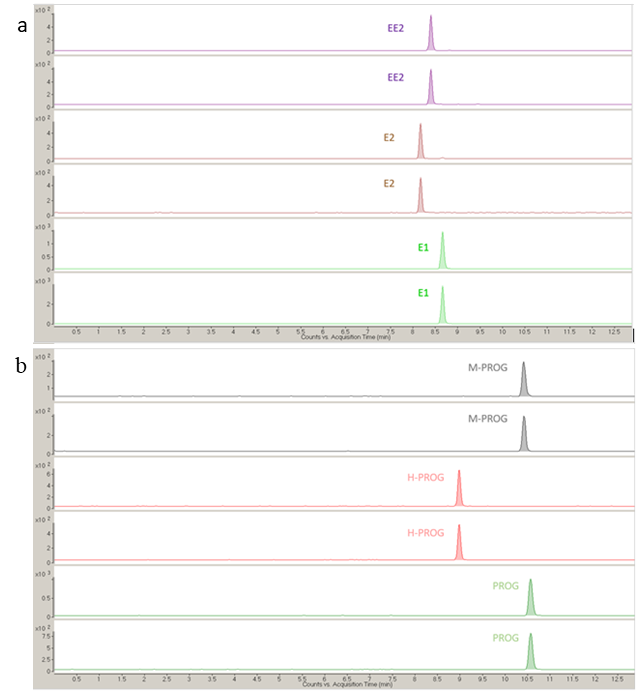


**Appendix 2: Water samples**

**Study Area**

The lakes are located south of the Alps barrier, in the central-eastern hydrographic basin of the Po River, one of the highest populated and industrialized European area with an agriculture and farming vocation. Additionally, these lakes are an important resource for recreation and tourism from Italy and Europe. Garda Lake is the largest lake in Italy, with an average surface area of 370 km^2^, belonging to three administrative regions, i.e., Lombardy, Veneto and Trentino-Alto Adige. The long tourist tradition brings an average of 20 million visitors a year, mostly international, to the shores of the lake, which can also have a direct influence on water quality (Binelli et al. 2020; Galafassi et al. 2021; Marziali et al. 2021; Hinegk et al. 2022). Como Lake is the deepest lake in Italy and the third in terms of surface area and volume. Its unusual shape of an inverted Y makes it a peculiar case of water cycle, since there is no effluent in the southwestern branch, thus the water moves out of the bay only through lake currents (Castiglioni et al. 2020). Mergozzo Lake is a small lake, which in the past represented the extreme tip of the western arm of Lake Maggiore (Marziali et al. 2021). The waters of this lake have been declared among the purest and cleanest in Italy due to the lack of industries on the shores, the ban on the use of motor boats and a sewerage system which does not discharge into the basin. Therefore, in this study it has been selected as blank lake sample for the method development. The sampling points were close to the following cities: Sirmione (Lombardy) for Garda Lake, Cernobbio (Lombardy) for Como Lake and Mergozzo (Piedmont) for Mergozzo Lake.

Tanaro and Ticino are two important rivers of Northern Italy, both tributaries of Po River. Tanaro River has origin in the Maritime Alps (Liguria) and is the only right bank tributary with high average discharge (Marchina et al. 2015; Balestrini et al. 2021; Winkler et al. 2022). Tanaro was sampled in the city of Alessandria (Piedmont). Ticino River rises in Switzerland and later flows through Lake Maggiore. Samples from Ticino River were collected in three different sampling points, Pavia city center, upstream and downstream.

References:

Balestrini R, Delconte CA, Sacchi E, Buffagni A (2021) Groundwater-dependent ecosystems as transfer vectors of nitrogen from the aquifer to surface waters in agricultural basins: The fontanili of the Po Plain (Italy). Sci Total Environ 753:141995. https://doi.org/10.1016/j.scitotenv.2020.141995

Binelli A, Pietrelli L, Di Vito S, et al (2020) Hazard evaluation of plastic mixtures from four Italian subalpine great lakes on the basis of laboratory exposures of zebra mussels. Sci Total Environ 699:134366. https://doi.org/10.1016/j.scitotenv.2019.134366

Castiglioni S, Zuccato E, Fattore E, et al (2020) Micropollutants in Lake Como water in the context of circular economy: A snapshot of water cycle contamination in a changing pollution scenario. J Hazard Mater 384:121441. https://doi.org/10.1016/j.jhazmat.2019.121441

Galafassi S, Sighicelli M, Pusceddu A, et al (2021) Microplastic pollution in perch (Perca fluviatilis, Linnaeus 1758) from Italian south-alpine lakes. Environ Pollut 288:117782. https://doi.org/10.1016/j.envpol.2021.117782

Hinegk L, Adami L, Zolezzi G, Tubino M (2022) Implications of water resources management on the long-term regime of Lake Garda (Italy). J Environ Manage 301:113893. https://doi.org/10.1016/j.jenvman.2021.113893

Marchina C, Bianchini G, Natali C, et al (2015) The Po River water from the Alps to the Adriatic Sea (Italy): new insights from geochemical and isotopic (δ18O-δD) data. Environ Sci Pollut Res 22:5184–5203. https://doi.org/10.1007/s11356-014-3750-6

Marziali L, Guzzella L, Salerno F, et al (2021) Twenty-year sediment contamination trends in some tributaries of Lake Maggiore (Northern Italy): relation with anthropogenic factors. Environ Sci Pollut Res 28:38193–38208. https://doi.org/10.1007/s11356-021-13388-6

Winkler A, Antonioli D, Masseroni A, et al (2022) Following the fate of microplastic in four abiotic and biotic matrices along the Ticino River (North Italy). Sci Total Environ 823:153638. https://doi.org/10.1016/j.scitotenv.2022.153638

**Table S2.1.** Physical-chemical parameters of the water samples used for method development

|  | Tap Water | Mergozzo Lake | Downstream UWWTP effluent |
| --- | --- | --- | --- |
| pH | 7.7 | 7.8 | 7.1 |
| χ (μS cm^-1^) | 270 | 59 | 333 |
| HCO_3_^-^ (mg L^-1^) | 184 | n.a. | 63 |
| Cl^-^ (mg L^-1^) | 5.0 | 2.2 | 40 |
| NO_3_^-^ (mg L^-1^) | 0.6 | 3.0 | 58 |
| SO_4_^2-^ (mg L^-1^) | 5.0 | 6.0 | 22.6 |
| Ca^2+^ (mg L^-1^) | 36.1 | 7.0 | 25.3 |
| Mg^2+^ (mg L^-1^) | 8.1 | 1.6 | 7.3 |
| Na^+^ (mg L^-1^) | 12.1 | 2.7 | 34 |
| K^+^ (mg L^-1^) | 1.5 | n.a. | 7.1 |
| SiO_2_ (mg L^-1^) | 17.5 | n.a. | 16.5 |

n.a.=not available

**Table S2.2.** Physical-chemical parameters of water samples used for monitoring campaign.

|  | Como Lake | Garda  Lake | Ticino River (city centre) | Ticino River (upstream) | Ticino River (downstream) | Tanaro River | UWWTP effluent pre- UV | UWWTP effluent post- UV |
| --- | --- | --- | --- | --- | --- | --- | --- | --- |
| pH | 7.8 | 7.1 | 7.6 | 7.2 | 7.4 | 7.9 | 7.1 | 7.1 |
| χ (μS cm^-1^) | 299 | 278 | 219 | 166 | 178 | 354 | 333 | 333 |
| HCO_3_^-^ (mg L^-1^) | 244 | n.a. | n.a. | 74 | 86 | n.a. | 63 | 63 |
| Cl^-^ (mg L^-1^) | 7 | 13.3 | 8.9 | 5.2 | 5.3 | 14.5 | 40 | 40 |
| NO_3_^-^ (mg L^-1^) | 4 | 9.0 | 7.2 | 2.3 | 3.8 | 7.2 | 58 | 58 |
| SO_4_^2-^ (mg L^-1^) | 15 | 14.3 | 25.5 | 23.2 | 23.9 | 40.8 | 22.6 | 22.6 |
| Ca^2+^ (mg L^-1^) | 49 | 39 | 30.3 | 25.0 | 27.3 | 58 | 25.3 | 25.3 |
| Mg^2+^ (mg L^-1^) | 10 | 7.2 | 6.0 | 5.7 | 6.0 | 9.4 | 7.3 | 7.3 |
| Na^+^ (mg L^-1^) | 9 | 9.5 | n.a. | 4.8 | 5.1 | n.a. | 34 | 34 |
| K^+^ (mg L^-1^) | 2 | n.a. | n.a. | 1.3 | 1.4 | n.a. | 7.1 | 7.1 |
| SiO_2_ (mg L^-1^) | n.a. | n.a. | n.a. | 2.2 | 3.6 | n.a. | 16.5 | 16.5 |

**Appendix 3: Analytical results**

**Table S3.1.** Matrix-matched calibration curves, correlation coefficients, matrix effect in lake water sample.

|  | Calibration equation* | R^2^ | ME % |
| --- | --- | --- | --- |
| E2 | y= 231(11) + 151(409) | 0.9916 | 20 |
| EE2 | y= 276(11) + 192(442) | 0.9931 | 10 |
| E1 | y= 1861(79) + 1041(3033) | 0.9929 | 14 |
| PROG | y= 2668(111) + 5578(4255) | 0.9932 | -61 |
| M-PROG | y= 1078(53) + 1890(2043) | 0.9904 | -61 |
| H-PROG | y= 422(17) + 94(648) | 0.9932 | -61 |

***** Calculated as peak areas vs. concentration; in parentheses the uncertainties from OLLSR and associated to slope and intercept of the mean calibration line.

**Table S3.2.** Matrix-matched calibration curves, correlation coefficients, matrix effect in simulated river water.

|  | Calibration equation* | R^2^ | ME % |
| --- | --- | --- | --- |
| E2 | y= 172(2) + 103(76) | 0.9996 | -11 |
| EE2 | y= 212(4) + 108 (159) | 0.9990 | -16 |
| E1 | y= 1391(19) + 1067(783) | 0.9994 | -15 |
| PROG | y= 3427(37) + 2996(1504) | 0.9996 | -49 |
| M-PROG | y= 1912 (35) + 1969(1423) | 0.9990 | -33 |
| H-PROG | y= 896(17) - 507(676) | 0.9990 | -58 |

***** Calculated as peak areas vs. concentration; in parentheses the uncertainties from OLLSR and associated to slope and intercept of the mean calibration line.

**Table S3.3.** Matrix-matched calibration curves, correlation coefficients, matrix effect in effluent UWWTP sample.

|  | Calibration equation* | R^2^ | ME % |
| --- | --- | --- | --- |
| E2 | y= 183(5) + 100(211) | 0.9964 | -5 |
| EE2 | y= 227 (9) + 195(364) | 0.9931 | -10 |
| E1 | y= 1741(33) - 184 (1283) | 0.9985 | 7 |
| PROG | y= 3645(68) - 1479(2623) | 0.9986 | -46 |
| M-PROG | y= 2065(32) - 46(1231) | 0.9990 | -27 |
| H-PROG | y= 979(67) - 1940(2565) | 0.9987 | -54 |

***** Calculated as peak areas vs. concentration; in parentheses the uncertainties from OLLSR and associated to slope and intercept of the mean calibration line.
